# Increased plasma and brain immunoglobulin A in Alzheimer’s disease is lost in apolipoprotein E *ε4* carriers

**DOI:** 10.1186/s13195-022-01062-z

**Published:** 2022-08-26

**Authors:** Dovilė Pocevičiūtė, Cristina Nuñez-Diaz, Bodil Roth, Shorena Janelidze, The Netherlands Brain Bank, Andreas Giannisis, Oskar Hansson, Malin Wennström

**Affiliations:** 1 Clinical Memory Research Unit, Department of Clinical Sciences Malmö, Lund University, Malmö, Sweden; 2 Department of Internal Medicine, Lund University, Skåne University Hospital, Malmö, Sweden; 3 Netherlands Institute for Neuroscience, Meibergdreef 47, 1105 BA Amsterdam, The Netherlands; 4 Department of Biochemistry and Biophysics, Stockholm University, Stockholm, Sweden; 5 Memory Clinic, Skåne University Hospital, Malmö, Sweden

**Keywords:** Amyloid beta, Blood-brain barrier, Immunoglobulin, Inflammation

## Abstract

**Background:**

Alzheimer’s disease (AD) is foremost characterized by β-amyloid (Aβ)-extracellular plaques, tau-intraneuronal fibrillary tangles (NFT), and neuroinflammation, but over the last years it has become evident that peripheral inflammation might also contribute to the disease. AD patients often demonstrate increased levels of circulating proinflammatory mediators and altered antibody levels in the blood. In our study, we investigated the plasma Immunoglobulin A (IgA) levels in association with apolipoprotein E (*APOE*) *ε4* status and Aβ pathology.

**Methods:**

IgA levels in antemortem-collected (cohort I) and postmortem-collected (cohort II) plasma samples from AD patients (*n* = 30 in cohort I and *n* = 16 in cohort II) and non-demented age-matched controls (NC) (*n* = 42 in cohort I and *n* = 7 in cohort II) were measured using ELISA. Hippocampal sections from cohort II were immunostained against IgA, and the IgA area fraction as well as the number of IgA positive (IgA+) cells in the cornu ammonis region were analysed using ImageJ. The relationship between plasma IgA levels and cognition, C-reactive protein (CRP), and cerebrospinal fluid (CSF) AD biomarkers in cohort I as well as neuropathology, IgA+ cell number, and IgA area fraction in cohort II was analysed before and after grouping the cohorts into *APOEε4* carriers and *APOEε4* non-carriers.

**Results:**

Plasma IgA levels were higher in AD patients compared to NC in both cohorts. Also, AD patients demonstrated higher IgA area fraction and IgA+ cell number compared to NC. When *APOEε4* status was considered, higher plasma IgA levels in AD patients were only seen in *APOEε4* non-carriers. Finally, plasma IgA levels, exclusively in *APOEε4* non-carriers, were associated with cognition, CRP, and CSF Aβ levels in cohort I as well as with IgA area fraction, IgA+ cell number, and Aβ, Lewy body, and NFT neuropathology in cohort II.

**Conclusions:**

Our study suggests that AD pathology and cognitive decline are associated with increased plasma IgA levels in an *APOE* allele-dependent manner, where the associations are lost in *APOEε4* carriers.

**Supplementary Information:**

The online version contains supplementary material available at 10.1186/s13195-022-01062-z.

## Background

Alzheimer’s disease (AD) is a neurodegenerative disorder characterized by β-amyloid (Aβ)-containing extracellular plaques and tau-containing intracellular neurofibrillary tangles (NFT) [1, 2]. However, AD is also associated with inflammatory processes both within the brain and in the periphery. The inflammatory processes in the brain are foremost characterized by the activation of glial cells and increased production of pro-inflammatory mediators, which is reflected in the cerebrospinal fluid (CSF) [3, 4]. Associations between the peripheral inflammation and AD can be found in epidemiological studies demonstrating a decreased risk of AD in individuals using non-steroidal anti-inflammatory drugs [5] and an increased risk of AD in individuals with, for instance, altered gut microbial composition [6], long-term exposure to air pollution [7, 8], and oral infections [9]. Several studies also show altered levels of cytokines, such as interleukin (IL)-6 and IL-1, and complements in the blood of AD patients; however, other studies do not confirm these results [10]. To establish the link between AD and peripheral inflammation further, studies have investigated the impact of AD pathology on systemic antibody levels. Antibodies can be found in five different isotypes, from which the most prominent isotypes are immunoglobulin G (IgG), M (IgM), and A (IgA). In the primary immune response, IgM is the first antibody isotype being produced during B cell development [11], whereas in the secondary immune response, mainly IgG is produced and in smaller amounts also IgA. IgG is one of the most abundant proteins in human serum (70 to 160 g/l in serum), being produced in a delayed response to an infection [12, 13]. IgA, on the other hand, is not as abundant (7 to 40 mg/l in serum) and is found in two forms: monomeric in serum and dimeric in the mucosa (i.e. saliva, tears, colostrum, intestinal and genital tract, respiratory secretions) [12, 14]. Previous studies have analysed IgA antibody levels in the blood of AD patients and healthy age-matched controls, but the results are inconsistent with demonstrating either increased [15–18], unaltered [19, 20], or decreased [21] blood IgA levels in AD patients compared to healthy controls. Normally, circulating antibodies are thought to be largely excluded from the immune-privileged central nervous system (CNS) in healthy individuals. However, increased levels of IgA antibodies have been observed in the CSF of many neurological patients, including AD patients [20, 22–26]. Hence, it has been hypothesized that systemically produced antibodies enter the CSF from blood through the breaches in the blood-brain barrier (BBB) that result from pathological processes such as neuroinflammation [27]. For instance, Goldwaser et al. reported that IgG autoantibodies cross the BBB, bind to neuronal surface molecules, and enhance Aβ42 penetration and deposition into neuronal cells, possibly leading to subsequent neuronal dysfunction and loss of synapses [28]. Whether other antibody isotypes, such as IgA or IgM, also cross the BBB and bind to brain cells is less clear. An increasing number of studies report that the inflammatory response in the periphery is also associated with apolipoprotein E (*APOE*) gene [29–31], the main genetic determinant for late-onset AD. The APOE is produced in several organs (i.e. liver, adrenal gland, brain) and by various cell types (i.e. ovarian and adrenal cells, macrophages, astrocytes, oligodendrocytes, pericytes, choroid plexus cells, neurons) and is associated with lipid transport and cholesterol homeostasis [32]. There are three alleles of the *APOE* gene: *APOEε2*, *APOEε3*, and *APOEε4*, from which the latter increases the risk of AD by 3–4 times in heterozygotes and by 12–15 times in homozygotes compared with *APOEε3* carriers [33]. The role of APOEε4 in AD is mostly established in the CNS, where it is known to affect Aβ aggregation and clearance, as well as influence neuroinflammation, BBB permeability, synaptic plasticity, and tau hyperphosphorylation [34]. However, recent findings suggest a direct link between liver-derived APOEε4 and pathological changes in the mouse brain [35]. Additionally, given its suggested immune-modulatory effect [29–31], it may be that *APOEε4* contributes to AD pathology via its impact on the peripheral immune response. Such impact might explain the inconsistency in plasma IgA levels reported by other groups [15–21] as *APOE* polymorphism was not considered in previously published studies. Hence, we found it interesting to investigate the IgA levels in AD patients in general and when the *APOE* polymorphism is accounted for. We therefore analysed the plasma IgA levels in two cohorts consisting of AD patients and non-demented controls (NC), where the plasma was collected antemortem (cohort I) and postmortem (cohort II). To further analyse the potential effect of *APOEε4* on IgA levels in relation to AD pathology, we divided the cohorts based on *APOEε4* status and investigated the differences between clinical diagnoses as well as associations between plasma IgA levels and cognition, CRP, and CSF AD biomarkers in cohort I and neuropathology and brain IgA immunoreactivity in cohort II.

## Methods

### Individuals included in the study

The study was performed on antemortem collected plasma samples from (*n* = 72) individuals (cohort I) and postmortem collected plasma and brain samples from (*n* = 23) individuals (cohort II). Cohort I consisted of NC (*n* = 42) and AD (*n* = 30) patients examined at the Memory Clinic at Skåne University Hospital, Sweden. Both controls and AD patients underwent cognitive and neurological assessments by a physician with a special interest in dementia disorders. Patients with AD were diagnosed according to the DSM-IV Criteria for Alzheimer’s disease. The cognitively healthy individuals displayed no neurological or cognitive deficiency symptoms. None of the study participants from cohort I had a systemic illness or was using anti-inflammatory medications. The NC and AD patients have been included in a previous study, and thus, the demographic data, scores of cognitive tests, *APOEε4* status, and levels of CSF AD biomarkers, Q-Albumin, and C-reactive protein (CRP) have been published previously [36]. The cognitive tests include the Alzheimer’s Disease Assessment Scale–Cognitive Subscale (ADAS-Cog), A Quick Test (AQT), and Mini-Mental State Examination (MMSE). The CSF AD biomarkers Aβ40, Aβ42, phosphorylated tau (p-tau), and total tau (t-tau) were analysed using Euroimmun ELISA (Euroimmun AG), and albumin levels in plasma and CSF samples were measured by immunoturbidimetry on a Roche Cobas Analyzer (Roche Diagnostics). The Q-Albumin was calculated as CSF albumin (mg/l)/plasma albumin (g/l) ratio and was used as a measure of the BBB function. The mean values of the variables can be found in Table 1.

**Table 1:** Clinical data of individuals included in cohort I

	NC, *n* = 42	AD, *n* = 30
Age (years)	74 ± 6	74 ± 7^a^
Females (%)	69	70
*APOEε4* carriers (%)	38	70
ADAS-Cog (score)	1.88 ± 1.70	8.67 ± 1.90***^a^
AQT (score)	63.24 ± 10.36	110.18 ± 46.72***^a^
MMSE (score)	29.19 ± 0.83	19.50 ± 4.27***^a^
CRP (mg/l)	1.75 ± 1.59	6.94 ± 13.12*^a^
CSF Aβ40 (pg/ml)	6759.52 ± 1906.93	6452.66 ± 1675.20^a^
CSF Aβ42 (pg/ml)	791.67 ± 289.29	386.70 ± 110.29***^a^
CSF p-tau (pg/ml)	45.61 ± 18.16	120.40 ± 41.24***^a^
CSF t-tau (pg/ml)	341.53 ± 106.23	621.81 ± 207.42***^a^
Q-albumin	6.75 ± 3.80	6.80 ± 2.54^a^

*Aβ* amyloid beta, *AD* Alzheimer’s disease, *ADAS-Cog* Alzheimer’s Disease Assessment Scale-Cognitive Subscale, *APOE4* apolipoprotein 4, *AQT* A Quick Test, *CRP* C-reactive protein, *CSF* cerebrospinal fluid, *MMSE* Mini-Mental State Examination, *NC* non-demented control, *p-tau* phosphorylated tau, *t-tau* total tau

^a^Data were analysed using either Student’s *t*-test or Mann-Whitney *U*-test, and values are presented as mean value ± SD

*Significant at *p* ≤ 0.05 level

**Significant at *p* ≤ 0.01 level

***Significant at *p* ≤ 0.001 level

Cohort II consisted of donors from The Netherlands Brain Bank (NBB) and included NC (*n* = 7) and clinically verified AD patients (*n* = 16). The presence of Aβ plaques was scored into O, A, B, and C according to Braak [37], where O = zero, A = some, B = moderate, and C = many, and the presence of NFT and neuropil threads was scored according to Braak stages I–VI [37]. Demographic data of the NC and AD groups is shown in Table 2, and demographic data, neuropathological evaluation, and cause of death of each case are found in Supplementary Table 1. Five individuals (*n* = 1 NC and *n* = 4 AD patients) were treated with anti-inflammatory medications during their last 3 months prior to death, and none of the individuals used anti-inflammatory medications during the last 24 h. The informed consent for the use of plasma samples, brain sections, and/or clinical data for research purposes was obtained from all patients included in cohorts I and II or their next of kin. The study was approved by the ethics committee in Lund, Sweden, and all investigations were conducted in agreement with the Declaration of Helsinki [38]. The procedures for brain tissue collection were approved by the Medical Ethics Review Committee of VU Medical Centre in Amsterdam (The Netherlands).

**Table 2:** Clinical data of individuals included in cohort II

	NC, *n* = 7	AD, *n* = 16
Age (years)	78 ± 9	80 ± 11^a^
Females (%)	57	56
*APOEε4* carriers (%)	14	69
PMD (h)	6.11 ± 1.30	6.06 ± 1.25^a^

*AD* Alzheimer’s disease, *APOE4* apolipoprotein 4, *NC* non-demented control, *PMD* postmortem delay

^a^Data were analysed using Student’s *t*-test, and values are presented as mean value ± SD

*Significant at *p* ≤ 0.05 level

**Significant at *p* ≤ 0.01 level

***Significant at *p* ≤ 0.001 level

### Stratification of cohorts

Individuals from both cohorts were stratified into the following groups: NC, AD, *APOEε4* non-carriers, and *APOEε4* carriers. Individuals with genotypes *APOEε23* (*n* = 4 in cohort I and *n* = 2 in cohort II) and *APOEε33* (*n* = 31 in cohort I and *n* = 9 in cohort II) were stratified as *APOEε4* non-carriers. Individuals with *APOEε24* (*n* = 2 in cohort I) as well as *APOEε34* (*n* = 29 in cohort I and *n* = 9 in cohort II) and *APOEε44* (*n* = 6 in cohort I and *n* = 3 in cohort II) were stratified as *APOEε4* carriers.

### Immunohistochemistry

The hippocampi of all individuals included in cohort II were stained against IgA using immunohistochemistry according to the following protocol: immersion paraformaldehyde (PFA)-fixed brain samples were sectioned into 40-μm sections using a microtome and kept free-floating in antifreeze medium in −20°C. For immunostainings, the sections were rinsed three times with phosphate-buffered saline containing potassium (KPBS) and quenched for 30 min at room temperature (RT) with a quenching solution (10% peroxidase, 10% methanol, and 80% KPBS). Then, brain sections were rinsed three more times with KPBS. Next, the blocking solution consisting of 5% bovine serum albumin (BSA) in KPBS with 0.25% triton (KPBS+) was added to each sample, and the samples were left to incubate for 1 h at RT. Following the incubation, polyclonal rabbit anti-human IgA antibody conjugated to horseradish peroxidase (HRP) (REF P0216, DakoCytomation, Glostrup, Denmark) was added directly to the blocking solution at a dilution of 1:300, and the samples were left to incubate for 3 nights (approximately 72 h) in the dark at 4°C with agitation. Following the incubation, the brain sections were washed three times with KPBS+ and two times with KPBS. Then, in the flow hood, KPBS was replaced with 3,3′-diaminobenzidine (DAB) diluted 1:50 in KPBS, and the samples were left to incubate for 10 min. Following the incubation, hydrogen peroxide (H_2_O_2_) diluted 1:10 in KPBS was added to the samples with DAB, and the samples were left to incubate for 2 min. Then, the DAB and H_2_O_2_ were removed, and the samples were washed 3 times with KPBS. Later, the brain sections were placed on glass slides, dried, and dehydrated in the following order: 5 min in ddH_2_O, 5 min in 70% ethanol (EtOH), 10 min in 95% EtOH, 10 min in 99% EtOH, and 10 min in xylene. Finally, the brain sections were mounted with mounting medium (DPX) and coverslipped. Analysis of the IgA immuno-stained area fraction in cohort II was performed by acquiring three images (3 × 0.15 mm^2^) of the cornu ammonis (CA1) region using bright-field microscopy (Olympus BX41). This region was chosen due to a pronounced AD pathology (Aβ plaques and NFT). The brain areas were defined based on area-characteristic landmarks and images within each brain area were captured by selecting the same three areas in three sections (in total 9 images) from each individual. The images were analysed using the Fiji software (ImageJ) by applying an automated threshold. The values were averaged and presented as mean optical density (OD) area fraction (%). To investigate the distribution of IgA-positive (IgA+) cells between the analysed groups, IgA+ cells were counted with ImageJ blinded to the observer and the average number of IgA+ cells per section was calculated.

### Analysis of plasma IgA

The plasma IgA levels were measured using a commercially available Human IgA ELISA kit (Mabtech, Nacka Strand, Sweden) according to the manufacturer’s instructions. Shortly, randomized samples blinded to the experimenter were diluted 1:50000 in incubation buffer and applied (in duplicates) to optically clear 96-well flat bottom microplates (Nunc, Thermo Scientific), coated with capture mAb MT57 diluted to 2 μg/ml in PBS. Then, detection mAb MT20-ALP diluted 1:1000 in incubation buffer was applied. Finally, the plates were incubated with pNPP substrate, and the optical density was measured in an ELISA reader at a wavelength range of 405–650 nm (BioTek).

### APOE genotyping

*APOE* genotype in cohort II was determined by real-time polymerase chain reaction (PCR) using TaqMan® Universal PCR Master Mix No AmpErase® UNG (Applied Biosystems) and TaqMan® single nucleotide polymorphisms genotyping assays (Thermo Scientific) targeting the rs429358 and rs7412 variants of the *APOE* gene. Amplification was performed using the QuantStudio™ 5 Real-Time PCR System, 384-well (Applied Biosystems), and the results were analysed using the Thermo Fisher Cloud software. Prior to genotype assessment, DNA was extracted from brain samples using the QIAGEN DNeasy Blood & Tissue Kit according to the supplier’s guidelines. Following extraction, DNA concentration and quality were assessed using NanoDrop™ One/OneC Microvolume UV-Vis Spectrophotometer (Thermo Scientific).

### Statistical analyses

All statistical analyses were performed using the SPSS software (version 28.0.0.0). The Kolmogorov-Smirnov test was used to assess normal distribution. Normally distributed samples (age, CSF Aβ40 levels, and plasma IgA levels in cohort I and age, postmortem delay, NFT scores, and plasma IgA levels in cohort II) were analysed using Student’s *t*-test. Non-normally distributed samples (ADAS-Cog scores, AQT scores, MMSE scores, CRP levels, CSF Aβ42 levels, CSF p-tau levels, CSF t-tau levels, and Q-albumin in cohort I and Aβ plaque scores, LB scores, CA1 IgA+ cell number, and CA1 IgA area fraction in cohort II) were analysed using the Mann-Whitney *U*-test. Correlations between the investigated variables in both cohorts were performed using the 2-tailed Spearman’s correlation test. Correlations and differences were considered significant at *p* ≤ 0.05.

## Results

### Plasma IgA levels are higher in AD patients

#### Cohort I

**Fig. 1:**
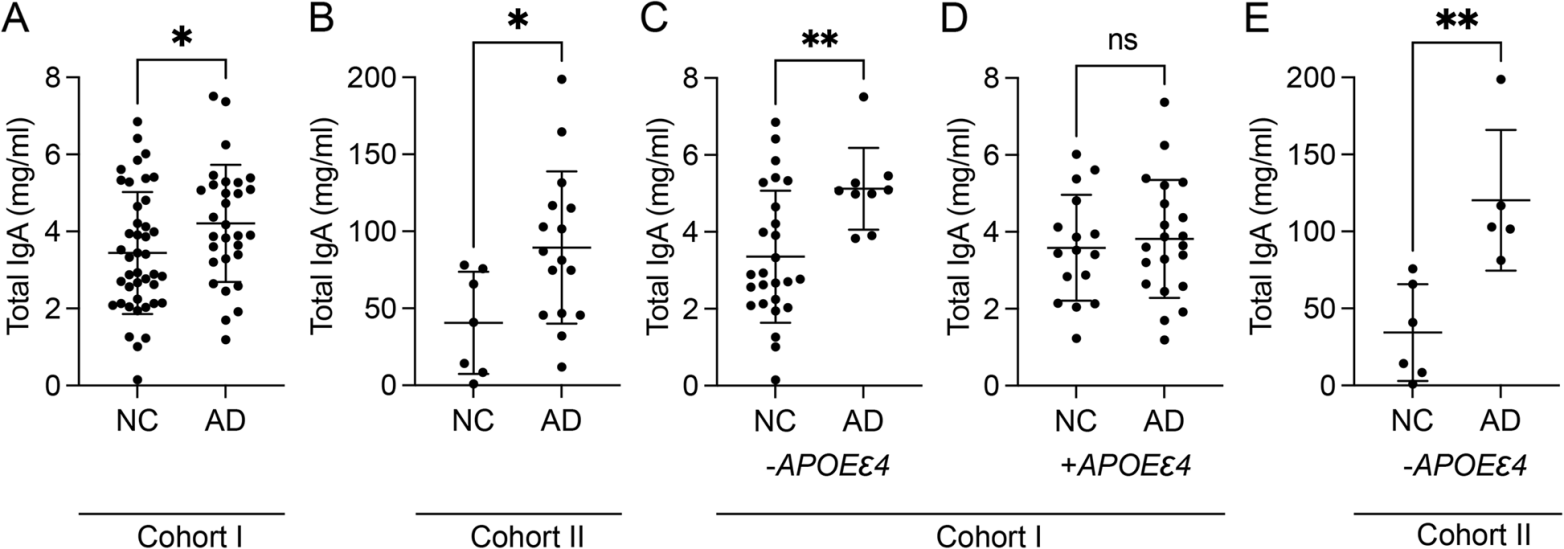
Plasma IgA levels in cohorts I and II. The graphs illustrate significantly higher IgA levels in AD patients compared to NC in cohort I (**A**) and cohort II (**B**), significantly higher IgA levels in AD patients compared to NC in *APOEε4* non-carriers (−*APOEε4*) in cohort I (**C**), unaltered IgA levels in AD patients compared to NC in *APOEε4* carriers (+*APOEε4*) in cohort I (**D**), and significantly higher IgA levels in AD patients compared to NC in *APOEε4* non-carriers in cohort II (**E**). Data were analysed using Student’s *t*-test and are presented as mean ± SD. ns, not significant. *Significant at *p* ≤ 0.05 level. **Significant at *p* ≤ 0.01 level. ***Significant at *p* ≤ 0.001 level

Analysis of plasma IgA levels showed that AD patients in cohort I had significantly higher IgA levels compared to NC (Fig. 1A). No significant differences in IgA levels were seen between males and females (4.14 ± 1.78 vs 3.60 ± 1.49 mg/ml, respectively) (*p* = 0.186).

#### Cohort II

In cohort II, plasma IgA levels were also significantly higher in AD patients compared to NC (Fig. 1B). As in cohort I, there was no significant difference in IgA levels between males and females (73.93 ± 51.45 vs 75.06 ± 50.82 mg/ml, respectively) (*p* = 0.959).

### Plasma IgA levels are higher in APOEε4 negative AD patients

#### Cohort I

Next, we analysed plasma IgA levels in *APOEε4* carriers and *APOEε4* non-carriers. In cohort I, the IgA levels did not differ between *APOEε4* carriers and non-carriers (3.72 ± 1.45 vs 3.81 ± 1.75 mg/ml, respectively) (*p* = 0.809). However, after the cohort was stratified into *APOEε4* carriers and non-carriers and the IgA levels were compared between NC and AD patients, the levels were significantly higher in AD patients compared to NC exclusively in *APOEε4* non-carriers (Fig. 1C). In contrast, there was no significant difference in IgA levels between *APOEε4*-carrying AD patients and NC (*p* = 0.637) (Fig. 1D). Furthermore, IgA levels in NC did not differ between *APOEε4* non-carriers and carriers (3.36 ± 1.72 vs 3.59 ± 1.38 mg/ml, respectively) (*p* = 0.652), but IgA levels in AD patients were significantly higher in *APOEε4* non-carriers compared to carriers (Additional file 2: Fig. S1).

#### Cohort II

In cohort II, there was also no significant difference in plasma IgA levels between *APOEε4* carriers and *APOEε4* non-carriers (75.64 ± 44.07 vs 73.39 ± 57.82 mg/ml, respectively) (*p* = 0.917). However, after the cohort was stratified into *APOEε4* carriers and non-carriers, the levels were significantly higher in AD patients compared to NC in *APOEε4* non-carriers (Fig. 1E). Of note, *APOEε4* carriers could not be analysed due to the low number of NC (*n* = 1) in this group. Furthermore, we noted higher, albeit not significantly, IgA levels in *APOEε4* non-carrying AD patients compared to *APOEε4*-carrying (120.26 ± 45.69 vs 75.43 ± 46.21 mg/ml, respectively) (*p* = 0.093).

### Hippocampal IgA area fraction is higher in AD patients

**Fig. 2:**
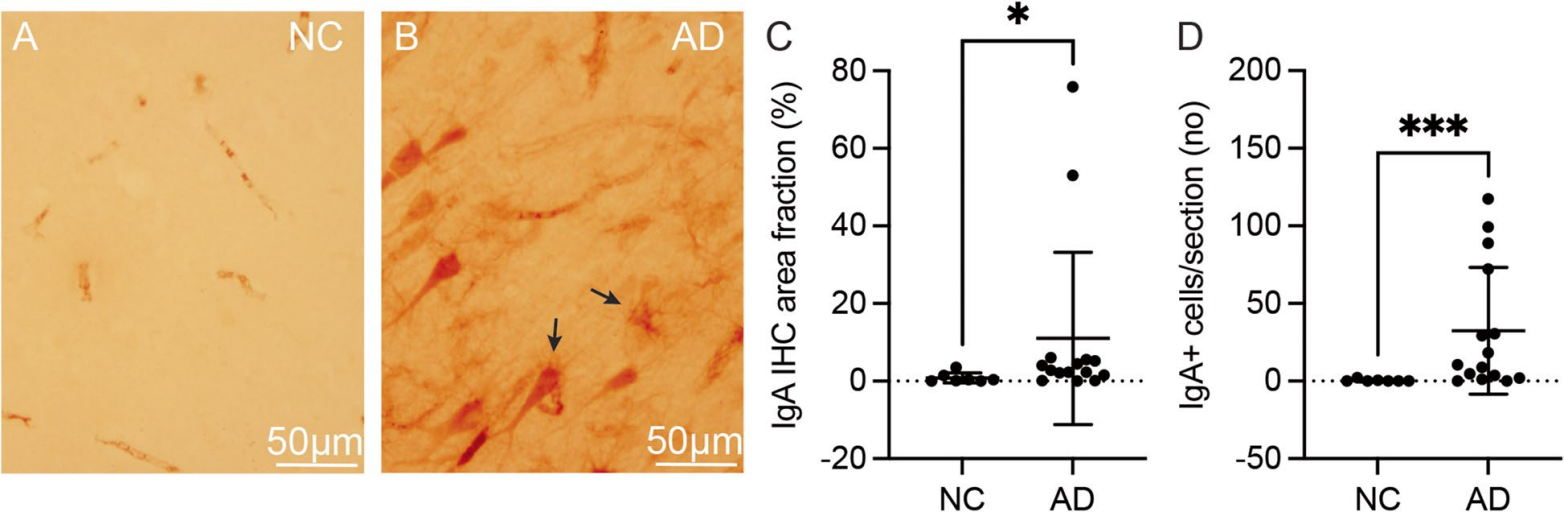
Hippocampal IgA immunoreactivity and IgA-positive cells. The images illustrate IgA immunostaining of the vessels in NC (**A**) and neurons as well as glial cells (both are indicated by black arrows) in AD patients (**B**). The graphs illustrate significantly higher IgA area fraction and IgA+ cell number in AD patients compared to NC (**C** and **D**, respectively). Data were analysed using the Mann-Whitney *U*-test and are presented as mean ± SD. *Significant at *p* ≤ 0.05 level. **Significant at *p* ≤ 0.01 level. ***Significant at *p* ≤ 0.001 level

The IgA area fraction in the hippocampal CA1 region was significantly higher in AD patients compared to NC (Fig. 2C). In contrast, the IgA area fraction did not differ between *APOEε4* non-carriers and carriers (8.03 ± 22.54 vs 7.54 ± 15.24, respectively) (*p* = 0.151). The IgA immunostaining yielded staining of vessels (Fig. 2A) and structures resembling neurons and glial cells (Fig. 2B). We found significantly more IgA+ cells in AD patients compared to NC (Fig. 2D). As in the case with IgA area fraction, there was no significant difference in the number of IgA+ cells between *APOEε4* carriers and non-carriers (30.75 ± 42.14 vs 13.74 ± 29.78, respectively) (*p* = 0.365). Based on the diagnosis, 29% of NC and 93% of AD patients had IgA+ cells. In the case of the *APOEε4* status, 64% of the *APOEε4* non-carriers and 82% of the carriers had IgA+ cells.

### Plasma IgA levels correlate with cognition, CRP, CSF AD biomarkers, neuropathology, and brain immunoreactivity

#### Cohort I

Finally, we performed correlation analyses in both cohorts. In cohort I, we analysed the correlation between the plasma IgA levels and cognition (assessed by ADAS-Cog, MMSE, and AQT memory tests), inflammation (indicated by CRP levels), BBB permeability (indicated by Q-albumin ratio), and CSF AD biomarkers such as CSF Aβ40, Aβ42, p-tau, and t-tau. When the whole cohort was analysed, IgA levels correlated with CSF Aβ40, CSF Aβ42, and CSF Aβ42/40 ratio (Table 3). When the cohort was stratified into NC and AD patients, IgA levels correlated with CSF Aβ42 only in NC (Table 3). When the cohort was stratified into *APOEε4* carriers and *APOEε4* non-carriers, the IgA levels correlated with MMSE, AQT, CRP, CSF Aβ40, CSF Aβ42, and CSF Aβ42/40 ratio only in *APOEε4* non-carriers (Table 3). Plasma IgA levels did not correlate with age in NC (*r* = − 0.086, *p* = 0.588).

**Table 3:** Correlations between the plasma IgA levels and cognition, CRP, and CSF AD biomarkers in cohort I

Variables	All groups	NC	AD	−*APOEε4*	+*APOEε4*
**MMSE (score)**	ns	ns	ns	− 0.499**	ns
**AQT (score)**	ns	ns	ns	0.438*	ns
**CRP (mg/l)**	ns	ns	ns	0.534**	ns
**CSF Aβ40 (pg/ml)**	− 0.246*	ns	ns	− 0.390*	ns
**CSF Aβ42 (pg/ml)**	− 0.349**	− 0.393*	ns	− 0.582***	ns
**CSF Aβ42/40**	− 0.268*	ns	ns	− 0.472**	ns

Data were analysed using Spearman’s correlation test

*Aβ* amyloid beta, *AD* Alzheimer’s disease, *APOE4* apolipoprotein 4, −*APOEε4 APOEε4* non-carrier, +*APOEε4 APOEε4* carrier, *AQT* A Quick Test, *CRP* C-reactive protein, *CSF* cerebrospinal fluid, *MMSE* Mini-Mental State Examination, *NC* non-demented control, *ns* not significant

*Significant correlation at *p* ≤ 0.05 level

**Significant correlation at *p* ≤ 0.01 level

***Significant correlation at *p* ≤ 0.001 level

#### Cohort II

In cohort II, we analysed the potential correlations between the plasma IgA levels and neuropathological features including Aβ plaques, Lewy bodies (LB), and NFT, as well as the IgA area fraction and IgA+ cell number in the hippocampal CA1 region. When the whole cohort was analysed, IgA levels correlated with IgA+ cell number and IgA area fraction (Table 4). When the cohort was divided upon diagnosis, IgA levels correlated with IgA area fraction only in AD patients (Table 4). When the cohort was stratified into *APOEε4* non-carriers and *APOEε4* carriers, IgA levels correlated with IgA+ cell number as well as Aβ, LB, and NFT neuropathology only in *APOEε4* non-carriers (Table 4). The IgA area fraction, on the other hand, correlated with IgA levels in both *APOEε4* non-carriers and carriers (Table 4). Plasma IgA levels did not correlate with age or postmortem delay in NC (*r* = 0.450, *p* = 0.310; *r* = − 0.432, *p* = 0.333, respectively).

**Table 4:** Correlations between the plasma IgA levels and neuropathology as well as hippocampal IgA area fraction and IgA-positive cell number in cohort II

Variables	All groups	NC	AD	−*APOEε4*	+*APOEε4*
**Aβ (score)**	ns	ns	ns	0.741**	ns
**LB (score)**	ns	ns	ns	0.702*	ns
**NFT (score)**	ns	ns	ns	0.840***	ns
**IgA+ cells (no.)**	0.560**	ns	ns	0.893***	ns
**IgA area fraction (%)**	0.688***	ns	0.564*	0.918***	0.618*

Data were analysed using Spearman’s correlation test

*Aβ* amyloid beta, *AD* Alzheimer’s disease, *APOE4* apolipoprotein 4, −*APOEε4 APOEε4* non-carrier, +*APOEε4 APOEε4* carrier, *IgA* immunoglobulin A, *IgA+* immunoglobulin A-positive, *LB* Lewy body, *NC* non-demented control, *NFT* neurofibrillary tangle, *no.* number, *ns* not significant

*Significant correlation at *p* ≤ 0.05 level

**Significant correlation at *p* ≤ 0.01 level

***Significant correlation at *p* ≤ 0.001 level

Subsequently, we analysed whether the IgA area fraction and the number of IgA+ cells in hippocampal CA1 correlate with Aβ plaques, LB, and NFT. When we analysed all groups, IgA area fraction correlated with Aβ score and IgA+ cell number correlated with both Aβ and NFT scores (Table 5). When we divided the cohort upon diagnosis, IgA+ cell number correlated with LB score only in NC (Table 5). Finally, when we looked at *APOEε4* non-carriers and *APOEε4* carriers, both IgA area fraction and IgA+ cell number correlated with Aβ, LB, and NFT scores exclusively in *APOEε4* non-carriers (Table 5).

**Table 5:** Correlations between the hippocampal IgA area fraction and IgA-positive cell number and neuropathology in cohort II

	Neuropathology (score)	All groups	NC	AD	−*APOEε4*	+*APOEε4*
**IgA area fraction (%)**	**Aβ**	0.489*	ns	ns	0.836***	ns
	**LB**	ns	ns	ns	0.735*	ns
	**NFT**	ns	ns	ns	0.855***	ns
**IgA+ cells (no.)**	**Aβ**	0.502*	ns	ns	0.862***	ns
	**LB**	ns	0.820*	ns	0.660*	ns
	**NFT**	0.506*	ns	ns	0.844***	ns

Data were analysed using Spearman’s correlation test

*Aβ* amyloid beta, *AD* Alzheimer’s disease, *APOE4* apolipoprotein 4, *−APOEε4 APOEε4* non-carrier, +*APOEε4 APOEε4* carrier, *IgA* immunoglobulin A, *IgA+* immunoglobulin A-positive, *LB* Lewy body, *NC* non-demented control, *NFT* neurofibrillary tangle, *no.* number, *ns* not significant

*Significant correlation at *p* ≤ 0.05 level

**Significant correlation at *p* ≤ 0.01 level

***Significant correlation at *p* ≤ 0.001 level

## Discussion

In the current study, we found increased levels of plasma IgA in AD patients, which was exclusively seen in *APOEε4* non-carriers. In addition, plasma IgA levels were associated with cognitive decline, CRP, Aβ pathology, and brain IgA immunoreactivity in *APOEε4* non-carriers. These associations were lost in *APOEε4* carriers.

The increase in plasma IgA levels in AD patients fits well with previous studies demonstrating higher blood IgA levels in AD patients [16–18] or patients with cognitive dysfunctions [15], but contradicts other studies showing unaltered [19, 20] or decreased [21] blood IgA levels in AD patients. However, it should be noted that none of the previous studies has accounted for *APOEε4* status. We show that the AD-related increase in plasma IgA levels is foremost attributed to *APOEε4* non-carriers. Thus, it may be that the inconsistency in the results of previous reports on blood IgA levels in AD patients is due to the varied inclusion of *APOEε4* carriers in analysed cohorts. The increase in plasma IgA levels in AD patients is interesting from the perspective that IgA is the most prominent Ig isotype found on mucosal surfaces, such as saliva, tears, colostrum, intestinal and genital tract, and respiratory secretions. In fact, globally, there is more IgA produced than all other isotypes combined [14, 39]. Thus, it is tempting to speculate that the increase in plasma IgA levels in AD patients is related to the inflammatory events in the mucosa previously reported to be associated with AD [40, 41]. However, the majority of IgA in the serum is monomeric (which is produced in the bone marrow), and only 1.6–7.1% of the total serum IgA is dimeric IgA deriving from the mucosa [14, 42]. Thus, we find it unlikely that the increase in plasma IgA levels in AD patients is due to a mucosa-related inflammation, but further studies are highly warranted.

Not only plasma IgA levels were higher in AD patients, but also a higher brain IgA area fraction and IgA+ cell number were detected in these individuals. The IgA immunostaining in NC was foremost seen in the vessels, while in AD patients, it was often found in the brain parenchyma where it was visualized as structures resembling neurons and glial cells. Other studies have demonstrated a significant increase in vessel-associated Igs in the parenchyma of AD brains compared to NC brain tissue. In addition, Ig-positive (Ig+) staining extended throughout the neuronal cell bodies, which showed apoptotic features that were not observed in Ig-negative neurons [43]. Another study performed on brain-reactive serum antibodies reported a significantly greater percentage of Ig+ neurons in the brain regions (including the hippocampus) of healthy human brains incubated with the serum of AD patients compared to the serum of NC. The authors indicated that neuron-binding antibodies are abundant in the serum of AD patients and that they could be either the cause or the result of cerebral lesions routinely found in AD brains [44]. The findings were supported by other research groups, as they demonstrated that circulating IgG autoantibodies bind to dying neurons in the vicinity of the cortical lesions in the brains of adult rats, possibly to participate in the phagocytosis and removal of injured neurons [45]. The mentioned studies, including our own, point out that AD pathology is associated with a higher frequency of circulating Ig binding to parenchyma and especially to neuronal and glial cells. The literature on human brain IgA immunoreactivity is rather scarce, but studies have demonstrated IgA+ neurons and glial cells in children aged 3 to 7 months [46] and IgA deposition in the brain of a patient with gluten ataxia [47]. The underlying cause of the increase of IgA area fraction in the brain parenchyma of AD patients in our study warrants further investigation, but a probable scenario is that the BBB permeability is enhanced in these patients. In our study, we found no correlation between plasma IgA levels and Q-albumin ratio, and the Q-albumin ratio did not differ between NC and AD patients in cohort I. However, previous studies have demonstrated an increased Ig immunoreactivity and the presence of Ig+ neurons and glial cells in postmortem AD brains in association with BBB [43, 48–50]. Whether inflammatory events in the periphery, reflected by the increase in plasma IgA levels, induce the BBB permeability or if the AD-related neuroinflammatory events in the brain cause BBB leakage is still under debate. The former idea is supported by studies demonstrating that proinflammatory mediators from the blood can enter the CNS via neural or humoral pathways promoting a proinflammatory environment in the brain and subsequently inducing molecular changes that exacerbate neurodegeneration [51]. For instance, in the animal model of AD, peripheral administration of lipopolysaccharide increased BBB permeability, brain IL-6 levels, and sickness behaviour in amyloid precursor protein (APP)-transgenic mice, suggesting that peripheral inflammation might spread to the brain through permeable BBB and affect the severity of behavioural problems in AD APP-transgenic mice [52]. In view of this finding, it is interesting that we documented a positive correlation between plasma IgA levels and hippocampal CA1 IgA area fraction in AD patients, as it suggests that peripheral IgA-mediated inflammatory processes are associated with an enhanced influx of IgA through the BBB. In addition, plasma IgA levels correlated negatively with CSF Aβ (indicative of increased brain Aβ load) in cohort I and positively with Aβ plaques in cohort II, suggesting that brain Aβ accumulation is associated with the elevation of IgA levels in the blood and brain. This idea fits well with the previous study, demonstrating that peripheral intravenous IgG penetrates through the BBB and enhances microglia-mediated clearance of Aβ [53].

The plasma IgA levels did not differ significantly between *APOEε4* carriers and *APOEε4* non-carriers in either of the analysed cohorts. This was, however, only evident when the NC in the two *APOEε4* groups were compared, which indicates that under normal non-pathological conditions IgA production is not affected by the *APOEε4*. This finding is in line with studies demonstrating no significant difference in either blood IgA levels [54] or plasma IgG levels [55] between *APOEε4* and *APOEε3* mice. However, when we compared AD patients based on *APOEε4* status, plasma IgA levels were significantly higher in *APOEε4* non-carriers compared to *APOEε4* carriers. Hence, it seems like the normal IgA response to AD-related inflammatory events (as hypothesized) is disturbed in *APOEε4* carriers. Such *APOEε4*-dependent disturbance of IgA response linked to AD has not (to our knowledge) been reported before.

The idea that the normal IgA response is disturbed in *APOEε4* carriers was further highlighted by the found correlations after analysis of cohort I. Here, plasma IgA levels correlated significantly with CRP, Aβ pathology, and cognitive decline exclusively in *APOEε4* non-carriers. The lost association between CRP and IgA in *APOEε4* carriers again supports the idea that *APOEε4* affects the relationship between IgA levels and inflammatory processes, while the association between CSF Aβ and IgA levels in *APOEε4* non-carriers supports a role for IgA in AD pathology. The cognitive decline was assessed by three memory tests, MMSE (a 30-point test, where lower scores indicate more severe cognitive impairment), AQT (a test of cognitive speed, where higher scores correspond to a slower perception speed to visual stimuli and a lower rate of correct answering), and ADAS-Cog (cognition rating scale, where higher scores indicate greater cognitive and non-cognitive dysfunction). Since plasma IgA levels correlated negatively with MMSE and positively with AQT in *APOEε4* non-carriers, it suggests that plasma IgA levels are associated with impaired cognition in these individuals. This finding, to our knowledge, has not been reported before. However, a recent study demonstrated a cognitive decline in patients with high levels of IgA anti-*N*-methyl-*D*-aspartate receptor autoantibodies [56], which fits well with our results.

Finally, it is important to point out that our study has limitations. Firstly, the sample size of both cohorts is small, in particular cohort II, which could contribute to statistical error type 1. Follow-up studies, larger cohorts (preferably with more *APOEε4* carriers in the postmortem NC group), are thus warranted to verify our result. We would however like to point out that although the sample sizes are small, we found similar results in both cohorts (i.e. increased levels of IgA in AD patients and associations between AD pathology and IgA exclusively in *APOEε4* non-carriers). Secondly, cohort II contained five cases (*n* = 1 NC and *n* = 4 AD patients) who were treated with anti-inflammatory medications during their last 3 months prior to death. The plasma IgA levels of the four AD cases did not differ from AD cases without anti-inflammatory treatment (data not shown), suggesting a minor impact of the medications in these cases. Nevertheless, we cannot entirely exclude the possibility that the anti-inflammatory medications (or the condition they are prescribed for) can affect the results.

To conclude, our study, demonstrating an association between plasma IgA levels and cognitive decline as well as AD pathology supports the idea that peripheral inflammation is implicated in AD. However, this event appears to be strongly affected by *APOEε4*, where the increase in plasma IgA levels and correlation with cognitive decline and AD pathology is lost in *APOEε4* carriers. These findings support the previously shown role for *APOEε4* in AD pathogenesis and highlight the heterogeneity of the disease, where polymorphic genetic factors modulating inflammatory responses might be of importance. The study further suggests that *APOEε4* should be accounted for when measuring plasma IgA levels, in particular in AD patients, which limits a potential application in clinical routine. Future studies investigating the impact of *APOEε4* on IgA production and regulation are warranted as they might shed a light on mechanisms implicated in the increased risk for AD in *APOEε4* carriers.

## Supplementary Information

**Additional file 1: Table S1.** Demographic data and neuropathological assessment of individuals included in cohort II.

**Additional file 2: Fig. S1.** Plasma IgA levels in patients in cohort I. The graph demonstrate significantly higher IgA levels in *APOEε4* non-carrying AD patients compared to *APOEε4*-carrying. Data were analysed using Student’s t-test and is presented as mean ± SD. ns – not significant. * Significant at *p* ≤ 0.05 level. ** Significant at *p* ≤ 0.01 level. *** Significant at *p* ≤ 0.001 level.

## Data Availability

The data sets supporting the conclusions of this article can be made available upon request. MDCS data can be requested through an application to the MDCS steering committee.

## Abbreviations

AD: Alzheimer’s disease
ADAS-Cog: Alzheimer’s Disease Assessment Scale-Cognitive Subscale
APOE: Apolipoprotein E
APP: Amyloid precursor protein
AQT: A Quick Test
Aβ: Amyloid beta
BBB: Blood-brain barrier
CA1: Cornu ammonis
CRP: C-reactive protein
CSF: Cerebrospinal fluid
Ig: Immunoglobulin
IgA: Immunoglobulin A
LB: Lewy body
MMSE: Mini-Mental State Examination
NC: Non-demented control
NFT: Neurofibrillary tangle
p-tau: Phosphorylated tau
PMD: Postmortem delay
t-tau: Total tau
