# Supplementary figures and images for: Increased plasma and brain immunoglobulin A in Alzheimer’s disease is lost in apolipoprotein E ε4 carriers
Source: Alzheimers Res Ther. 2022 Aug 26;14:117. doi: 10.1186/s13195-022-01062-z (PMC9414424; doi:10.1186/s13195-022-01062-z)

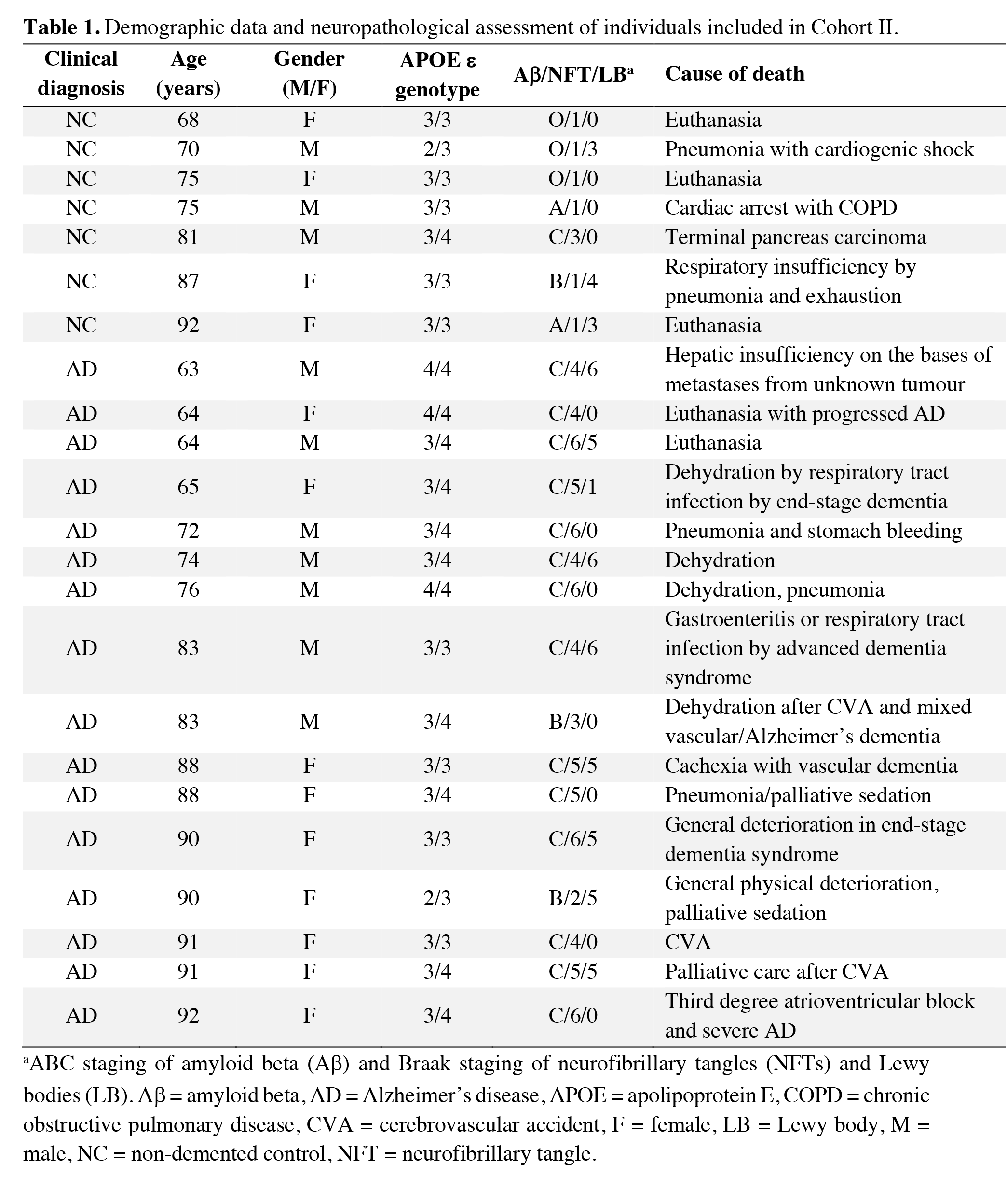

Supplement: Supplementary file 1 — Additional file 1: Table S1. Demographic data and neuropathological assessment of individuals included in cohort II. [file 13195_2022_1062_MOESM1_ESM.tif]

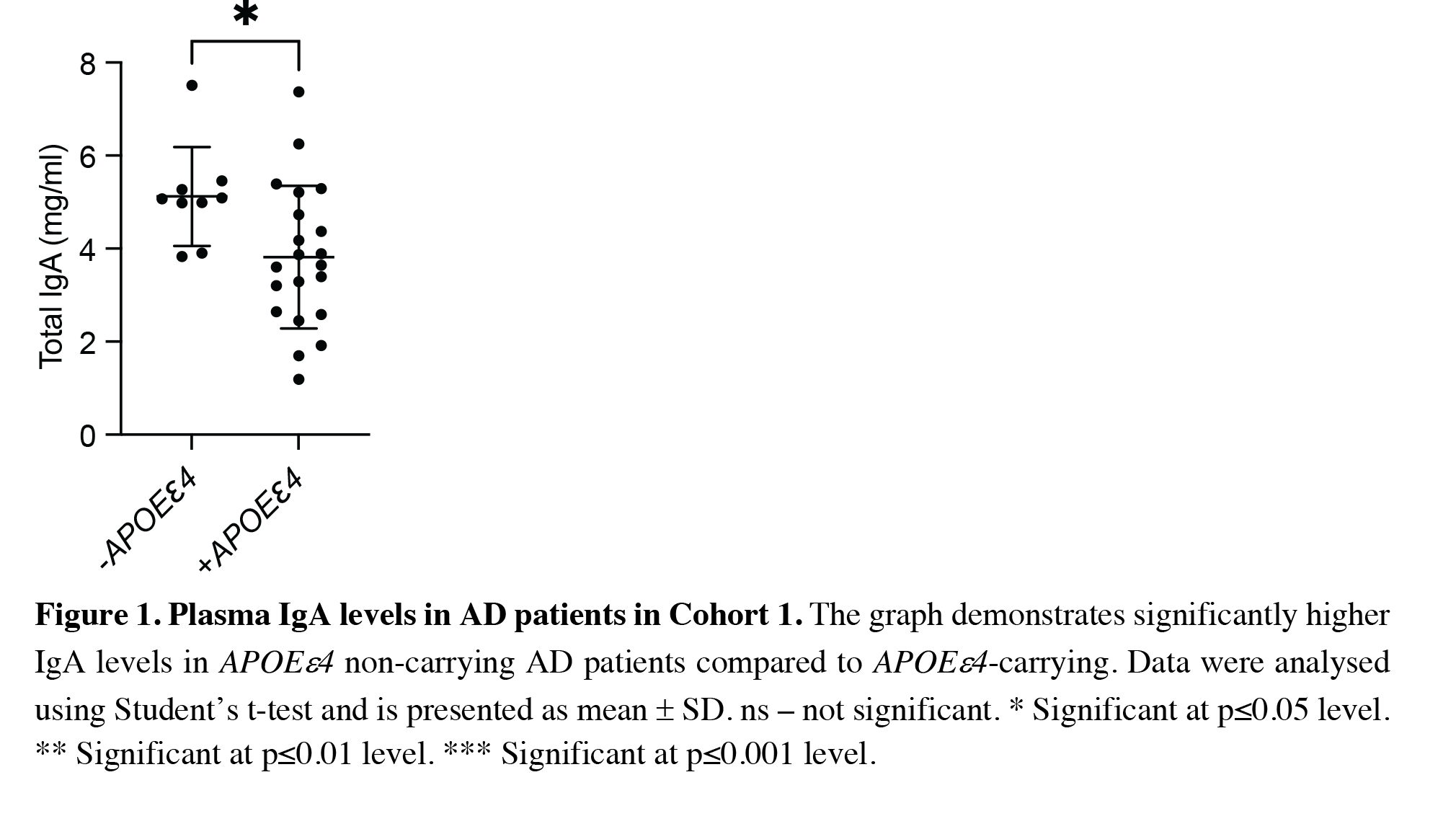

Supplement: Supplementary file 2 — Additional file 2: Fig. S1. Plasma IgA levels in patients in cohort I. The graph demonstrate significantly higher IgA levels in APOEε4 non-carrying AD patients compared to APOEε4-carrying. Data were analysed using Student’s t-test and is presented as mean ± SD. ns – not significant. * Significant at p ≤ 0.05 level. ** Significant at p ≤ 0.01 level. *** Significant at p ≤ 0.001 level. [file 13195_2022_1062_MOESM2_ESM.tif]
